# Mind the gap: an evidence gap analysis of digital literacy within public health systems in the European Union using two different frameworks

**DOI:** 10.3389/fpubh.2026.1868019

**Published:** 2026-08-18

**Authors:** Malin Siv Roppel, Marlene Lakemann, Dagmar Starke

**Affiliations:** 1 Department of Population Medicine and Health Services Research, School of Public Health, Bielefeld University, Bielefeld, Germany; 2 Academy of Public Health Services, Düsseldorf, Germany

**Keywords:** digital competence frameworks, digital literacy, essential public health functions, evidence gap analysis, public health services

## Abstract

This evidence gap analysis aimed to map the existing evidence and identify gaps in the literature on digital literacy among the public health workforce in the European Union. An evidence framework integrating the WHO’s Essential Public Health Functions and the European Commission’s DigiComp Framework 3.0 was applied to analyse literature identified via a structured search strategy. Twelve studies published between 2020 and 2025 were included. The findings reveal a fragmented and uneven evidence base. Research is heavily concentrated in the domain of public health workforce development, particularly in relation to communication and collaboration competencies, while several public health functions and digital competence areas remain underrepresented or unaddressed. Methodologically, the field is limited, with a clear lack of quantitative studies. Overall, the analysis highlights substantial gaps in both the conceptualisation and empirical investigation of digital literacy in public health. These findings underscore the need for more comprehensive, methodologically diverse research to support evidence-based workforce development and inform digital transformation strategies in European public health systems.

## 1 Introduction

European public health systems, like public administration more broadly, are undergoing profound digital transformation. In federal, multi-level public health systems (PHS) such as the German public health services, this transformation is especially complex because responsibilities are distributed across municipal, state, and federal levels, each with its own regulatory frameworks, stakeholders, and institutional priorities (1). As a result, each level faces distinct challenges, including unequal resources, divergent interests, and administrative barriers. Advancing digital transformation in this fragmented landscape therefore requires careful coordination and context-sensitive strategies. Despite these obstacles, digital transformation in the public health sector depends heavily on the expertise of the public health workforce, which must lead these changes. To do so, the workforce needs strong digital literacy and digital competencies (2–5).

Consequently, public health systems are affected from two directions. On the one hand, the public health workforce itself must be digitally literate; on the other hand, populations must possess digital literacy to effectively access and utilise public health services. This study focuses exclusively on the digital literacy and competencies of the public health workforce in the European Union.

Digital literacy is increasingly recognised as a fundamental competence across both public and private sectors. While the term is widely used in research and policy, its conceptual clarity and dimensional structure remain underdeveloped (6–9). The concept is frequently examined in various forms and under different synonyms. These include, for example, eGovernment competences, digital competences, digital maturity, and e-skills. Moreover, it is often conflated with the concept of digital health literacy (10–13).

In the context of the PHS, capacity building in digital literacy is a critical enabler of sustainable digital transformation as it includes the socio-technical perspective. In this paper, the public health workforce is understood as a diverse professional group working within PHS across the European Union. The public health workforce composition varies considerably across national contexts, for instance, in the German public health services, professionals such as social medical assistants (Sozialmedizinische Fachassistent:innen) combine prior clinical training with specialised public health education.

In this paper, digital literacy is defined as the ability to understand and use information in multiple formats from a wide range of digital sources, as originally described by Gilster (14), encompassing not only technical skills but also cognitive, social, creative, and ethical capacities, and functioning as an umbrella concept that integrates related literacies for effective participation in digital environments and socio-technical transformations. Differences in digital literacy levels between regions can exacerbate existing inequalities, positioning digital literacy as a critical variable in the context of social and health disparities. In an era of rapid digital transformation, the ability to engage with digital tools and infrastructures has become essential for maintaining employability, improving health outcomes, and implementing intersectoral strategies such as Health in All Policies (6). On a global scale, digital literacy has emerged as a key factor in national competitiveness (15, 16).

Digital competence, by contrast, refers to the specific operationalised skill sets required to engage in a digitised environment, the measurable, trainable components of digital literacy, and does not necessarily encompass the broader ability to drive digital transformation processes. For instance, the ability to evaluate information in digital environments to assess their credibility is a digital competence (17). Additionally, digital health literacy is a distinct but related concept, referring specifically to the ability to seek, find, understand, and appraise health information from electronic sources and apply that knowledge to address or solve a health problem (18). In the context of the public health workforce, digital health literacy represents a domain-specific application of broader digital literacy. While these three concepts overlap, they are not interchangeable and are used as distinct terms throughout this paper.

Several curricula and competency frameworks addressing digital literacy have already been developed, many of which are tailored to educational settings. For instance, the Health Information Technology Competency Framework (HITCOMP) outlines more than 1,000 competencies relevant to the health sector. The WHO-ASPHER Competency Framework for the Public Health Workforce in the European Region serves as guidance to not only strengthen public health workforce training but to support capacity-building, among others. Additionally, the European Commission’s Digital Competence framework (DigiComp) for citizens was recently renewed and amended (17). In addition, the WHO’s Essential Public Health Functions (EPHFs) define the core capacities required of the public health workforce and thus provide a structured framework for examining where and how digital literacy is needed in practice (19).

For the purposes of this analysis, the WHO EPHF framework and DigiComp were selected as the two analytical axes of the evidence matrix. Together they span a functional and a competency-based dimension, respectively. The EPHF framework defines what public health workforces do, while DigiComp operationalises what digital competencies look like in practice.

Several systematic reviews have explored the concept of digital literacy across various sectors, highlighting its multidimensional nature and contextual relevance (3, 4, 8, 9, 20). However, within the context of public health systems, research on digital literacy remains relatively scarce. To ground the digital transformation process in public health systems in the European Union in robust scientific evidence, the concept of digital literacy must first be clearly defined, and existing evidence gaps must be systematically identified and addressed.

This study therefore performs an evidence gap analysis and aims to examine where conceptual and empirical knowledge on digital literacy is lacking. Building on these findings, the study will formulate targeted recommendations to guide future research and policy. Ultimately, the outcomes of this analysis are intended to inform policy initiatives that strengthen digital literacy capacity among the public health workforce in the European Union.

## 2 Materials and methods

This study adopts a methodological approach informed by the PRISMA-Evidence and Gap Maps (EGM) framework according to Campbell et al. (21) as well as Miake-Lye et al. (22). The evidence gap map methodology was selected as it allows for the systematic visualisation of evidence distribution across a predefined two-dimensional framework, making both clusters of evidence and gaps identifiable. This distinguishes it from a scoping review, which characterises the breadth of a literature without necessarily mapping it against a structured analytical matrix.

Thereby, a three-step-methodological logic is followed. Firstly, the SPIDER framework (Sample, Phenomenon of Interest, Design, Evaluation, Research Type) (23) is used to define the conceptual scope, eligibility criteria and search strategy. Secondly, the PRISMA-EGM framework guided the screening and selection process and thirdly, a deductive coding matrix combining the WHO Essential Public Health Functions (EPHF 2024) and the European Digital Competence Framework (DigiComp) was used to categorise and map the included evidence.

### 2.1 Objective and scope

The objective of this evidence gap analysis is to identify existing evidence and map gaps concerning digital literacy of the public health workforce in Europe. In doing so, it aims to provide a foundation for informed decision-making and future capacity-building strategies. The scope of the review was defined using the SPIDER framework, which is particularly suitable for qualitative and mixed-methods research (23). As outlined in Table 1, the review focuses on the public health workforce in Europe, investigates digital literacy as the central phenomenon of interest, and includes published studies of any design. Eligible studies comprise qualitative, quantitative, mixed-methods approaches, and reviews.

**Table 1:** Research scope according to the SPIDER framework.

Spider framework dimensions	Research scope
Sample	Public Health Workforces in the European Union, Public Health Workers in the European Union
Phenomenon of Interest	Digital literacy, digital competency, digital skills, eHealth literacy
Design	Studies focused on public health settings, policies, or interventions
Evaluation	Studies evaluating digital literacy impact on workforce capacity, or training
Research Type	Qualitative, quantitative, evidence synthesis, reviews and mixed-methods peer-reviewed studies.

### 2.2 Evidence framework

Based on a rapid review of current literature on digital literacy within the context of public health, an evidence framework was developed to guide the identification and analysis of evidence gaps. This framework draws on the Essential Public Health Functions (EPHFs) as defined by the World Health Organization in 2024 (19) and the European Commission’s DigiComp Framework 3.0 (17). These two theoretical layers are integrated in a matrix, which serves both to structure the framework and to code the extracted data. Furthermore, both the EPHFs and the DigiComp Framework inform the development of the search strategy employed in this study.

### 2.3 Search strategy

The literature search was conducted by a single researcher across three electronic databases: PubMed, SCOPUS, Cochrane. The search terms were developed in four dimensions based on the SPIDER framework, which were systematically combined to construct a comprehensive search strategy. These dimensions and the specific terms used are detailed in Table 2. It presents the search blocks (A–F) used across all three databases. These blocks were developed by synthesising the conceptual scope defined through the SPIDER framework with the thematic dimensions of the two analytical frameworks and operationalising them into database-specific search term clusters. Table 3 outlines the tailored search strings applied to each individual database. The search strings were adapted to the indexing conventions of each database. The underlying search logic and conceptual scope were consistent throughout the strategies and based on the terms outlined in Table 2. The search strategy was refined iteratively in response to the relevance and volume of initial search results, ensuring a more targeted and comprehensive retrieval of pertinent literature.

**Table 2:** Search terms.

Dimension	Focus	Keywords, synonyms, and Boolean operators
A	Sample: Public Health Workforce	Public Health OR public health services OR community health services OR local health services OR local health authorities
B	Phenomenon of Interest: Digital Literacy	digital* OR Digi* OR eGov* OR digital literacy* OR digital competency OR digital competence OR eSkills OR eGovernment competence
C	Study Type: Published peer-reviewed studies	qualitative OR quantitative OR mixed methods OR reviews
D	Time Frame	(year: 2020–2025) OR (“since 2020”)
E	Public Health Function	“public health surveillance” OR “public health monitoring” OR “public health emergency management” OR “public health stewardship” OR “health planning” OR “health financing” OR “public health management” OR “health protection” OR “disease prevention” OR “early detection” OR “health promotion” OR “community engagement” OR “social participation” OR “public health workforce development” OR “health service quality” OR “health service equity” OR “public health research” OR “public health knowledge” OR “health product access” OR “health supplies” OR “health equipment” OR “health technologies”
F	Digital Competence Fields (DigiComp)	“information and data literacy” OR “digital communication” OR “digital collaboration” OR “digital content creation” OR “safety” OR “data protection” OR “problem solving” OR “eGovernment competence”

Search blocks (A–F) used across PubMed, Scopus, and Cochrane. The blocks were developed by synthesising the SPIDER framework components with the thematic dimensions of the WHO Essential Public Health Functions (EPHF) and the European Digital Competence Framework (DigiComp), and operationalising them into database-specific search term clusters.

**Table 3:** Search strategies.

Database	Search string	Hits (15.04.2026)
PubMed	(“public health workforce”[Title/Abstract] OR “public health services”[Title/Abstract] OR “community health services”[Title/Abstract] OR “local health services”[Title/Abstract] OR “local health authorities”[Title/Abstract] OR “health professionals”[Title/Abstract] OR “health personnel”[Title/Abstract] OR “health staff”[Title/Abstract]) AND ( digital*[Title/Abstract] OR digi*[Title/Abstract] OR “digital literacy”[Title/Abstract] OR “digital competence”[Title/Abstract] OR “digital competency”[Title/Abstract] OR “digital skills”[Title/Abstract] OR “ICT skills”[Title/Abstract] OR “computer literacy”[Title/Abstract] OR “eSkills”[Title/Abstract] OR “eHealth”[Title/Abstract] OR “eGovernment”[Title/Abstract] OR “digital transformation”[Title/Abstract] OR “digital readiness”[Title/Abstract]) AND (qualitative[Title/Abstract] OR quantitative[Title/Abstract] OR “mixed methods”[Title/Abstract] OR empirical[Title/Abstract] OR “case study”[Title/Abstract] OR review[Title/Abstract]) AND (“2020/01/01”[Date - Publication]: “2025/12/31”[Date - Publication])	916
SCOPUS	(TITLE-ABS-KEY (“public health workforce” OR “public health services” OR “community health services” OR “health professionals” OR “health personnel” OR “local health services” OR “local health authorities”)) AND (TITLE-ABS-KEY (“digital literacy” OR “digital competence” OR “digital skills” OR “ICT skills” OR “computer literacy” OR “digital transformation” OR “eGovernment”)) AND (TITLE-ABS-KEY (“training” OR “education” OR “capacity building” OR “preparedness” OR “implementation” OR “adoption” OR “intervention”)) AND (TITLE-ABS-KEY (qualitative OR quantitative OR “mixed methods” OR empirical OR review OR “case study”)) AND PUBYEAR > 2019 AND PUBYEAR < 2026	215
Cochrane	(“public health” OR “health workforce” OR “local health services”) AND (“digital” OR “technology” OR “ICT” OR “computer literacy”)	26

### 2.4 Screening and study selection

**Figure 1:**
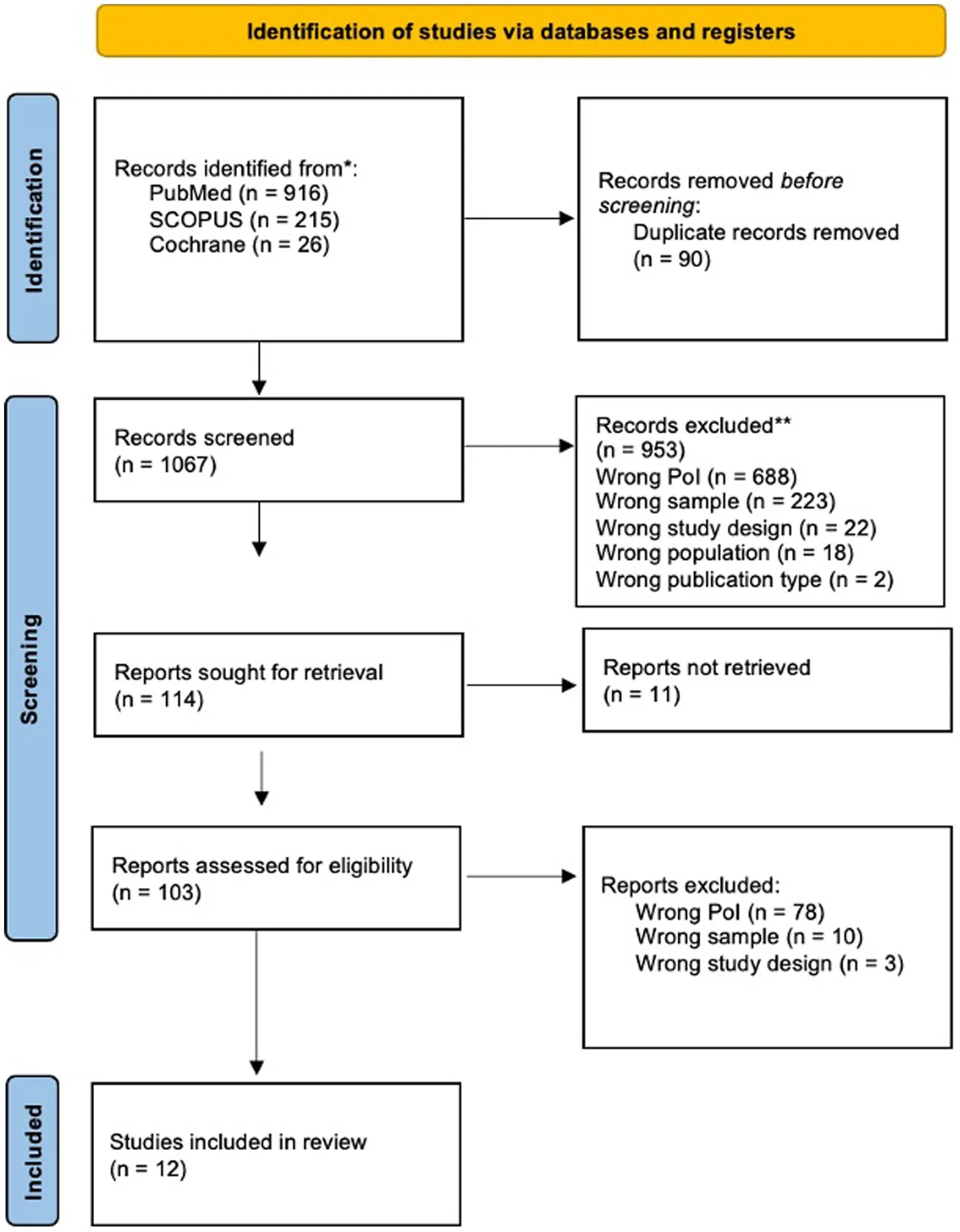
PRISMA 2020 flow diagram of the study selection process.

The screening process was conducted in a blinded manner by two researchers and consisted of two phases. In the initial phase, titles and abstracts were screened to exclude studies that did not meet the predefined inclusion and exclusion criteria (Table 4). Subsequently, full-text articles were assessed by two researchers to confirm eligibility for inclusion. Conflicts in inclusion decisions were resolved through discussions between two researchers. The software tool Rayyan was used to facilitate the screening process (24). The data extraction was conducted by a single researcher. The final coding was performed by two independent researchers using the EPHF and DigiComp framework definitions as deductive coding criteria. Each study was assigned to the EPHF function and DigiComp dimension whose definitions most closely corresponded to the study’s primary thematic focus. Discrepancies between coders were identified and resolved through discussion and consensus. The final coding results were verified by both researchers prior to matrix finalisation (Table 5). No formal quality appraisal of included studies was performed as the analysis focused on identifying the presence or absence of research rather than assessing effectiveness or synthesising study outcomes. The results of the screening and study selection process are presented in the PRISMA flow diagram shown in Figure 1.

**Table 4:** Eligibility criteria.

Framework	Definition	Inclusion criteria	Exclusion criteria
Sample	The population or group of interest	Workforces in healthcare, including public health workers and community health professionals in the European Union	Any other sample
Phenomenon of Interest	The topic, concept, or issue under study	Digital literacy, digital competency, digital skills, eHealth literacy	Any other concept not directly related to digital literacy
Design	The overall methodological approach of eligible studies (e.g., qualitative, quantitative, mixed-methods)	Studies focused on healthcare or public health settings, policies, or interventions	Any studies conducted in non-healthcare or unrelated contexts
Evaluation	Whether and how outcomes or competency levels are assessed within a study	Studies evaluating digital literacy impact on workforce capacity, or training.	Studies not measuring or addressing digital literacy
Research Type	The nature of the scholarly contribution (e.g., empirical study, review, evidence synthesis)	Qualitative, quantitative, or mixed-methods peer-reviewed studies	Non-peer-reviewed articles, irrelevant literature, or studies with unverified methodologies

**Table 5:** Evidence framework matrix (*X*-axis: dimensions of the digital competence framework for citizens, *Y*-axis: essential public health functions).

	Information search, evaluation and management	Communication and collaboration	Content creation	Safety, wellbeing and responsible use	Problem identification and solving
Public Health Surveillance and Monitoring	*N* = 0	*N* = 0	*N* = 0	*N* = 0	*N* = 0
Public Health Emergency Management	*N* = 0	*N* = 1 Interventional (*n* = 1) Rettinger et al. (35)	*N* = 0	*N* = 0	*N* = 1 Interventional (*n* = 1) Rettinger et al. (35)
Public Health Stewardship	*N* = 0	*N* = 0	*N* = 0	*N* = 0	*N* = 0
Multisectoral Planning, Financing and Management	*N* = 0	*N* = 0	*N* = 0	*N* = 0	*N* = 0
Health Protection	*N* = 0	*N* = 0	*N* = 0	*N* = 0	*N* = 0
Community Engagement and Social Participation	*N* = 0	*N* = 0	*N* = 0	*N* = 0	*N* = 0
Public Health Workforce Development	*N* = 2 Quantitative (*N* = 1) Mixed-Method (*N* = 1) Bigay et al. (25); Saigi-Rubio et al. (26)	*N* = 9 Qualitative (*n* = 7) Quantitative (*n* = 1) Mixed-Method (*n* = 1) Jarva et al. (27); Schumann et al. (28); Linares et al. (29); Chatzichristos et al. (30); Hammaren et al. (31); Bleijenbergh et al. (32); De Leeuw et al. (33); Bigay et al. (25); Saigi-Rubio et al. (26)	*N* = 1 Qualitative (*n* = 1) Schumann et al. (28)	0	*N* = 2 Qualitative (*n* = 2) Jarva et al. (27); Linares et al. (29)
Health Service Quality and Equity	*N* = 0	*N* = 2 Mixed-Method (*n* = 1) Qualitative (*n* = 1) Peres et al. (34); Burgos et al. (36)	*N* = 0	*N* = 0	*N* = 1 Mixed-Method (*n* = 1) Peres et al. (34)
Public Health Research, Evaluation and Knowledge	*N* = 0	*N* = 0	*N* = 0	*N* = 0	*N* = 0
Access to and Utilization of Health Products, Supplies and Technologies	*N* = 0	*N* = 4 Qualitative (*n* = 2) Mixed-Method (*n* = 1) Interventional (*n* = 1) Jarva et al. (27); Peres et al. (34); Rettinger et al. (35); Burgos et al. (36)	*N* = 0	*N* = 0	*N* = 3 Qualitative (*n* = 1) Mixed-Method (*n* = 1) Interventional (*n* = 1) Jarva et al. (27); Peres et al. (34); Rettinger et al. (35)

Evidence density: evidenced (*n* = 3 + studies), weakly evidenced (*N* = 1–2 studies), evidence gap (*n* = 0 studies).

Study design: qualitative, quantitative, mixed-method, interventional.

### 2.5 Data synthesis

Data synthesis was conducted in four structured steps. First, all eligible studies were transferred into a standardised data extraction table (Table 6). The following information was extracted: authors, year of publication, study type, geographical focus, aim of the study, digital competence area, essential public health function. In the second step, this information was categorised deductively using the previously developed evidence framework matrix. The matrix consisted of competence dimensions from the European Commission’s Digital Comp Framework (*X*-axis) and the EPHFs (*Y*-axis) outlined by the WHO. The third step involved developing an evidence gap map to visually represent the distribution of evidence and identify areas with limited or missing research. Finally, a set of recommendations for future research and practical application was derived based on the identified gaps.

**Table 6:** Data extraction.

#	Title	Authors	Year of publication	Study type	Geographical focus	Aim of the study	Digital competence area	Essential Public health function
1	Healthcare professionals’ perceptions of digital health competence: a qualitative descriptive study	Jarva et al. (27)	2022	Qualitative Study	Finland, Sweden	This study aims to provide insight into healthcare professionals’ lived experiences of digital health competence with the objective of improving the knowledge of how digital health competence is perceived by healthcare professionals.	Communication and Collaboration Problem Identification and solving	Access to and Utilization of Health Products, Supplies, Equipment and Technologies Public Health Workforce Development
2	Challenges of ICT use for nurse–patient communication in Portugal: a mixed methods research	Peres et al. (34)	2025	Mixed-Method Study	Portugal	The present study seeks to explore and clarify the specific knowledge, skills, and competencies that healthcare professionals, especially nurses, must develop to ensure effective communication with patients through digital means. The study investigates not only the contexts in which ICT is most beneficial or necessary but also the barriers faced in its adoption and the ethical, relational, and practical considerations involved. Through this inquiry, the aim is to generate scientific evidence that can inform the development of structured training programs and professional development strategies.	Communication and Collaboration Problem Identification and Solving	Access to and Utilization of Health Products, Supplies, Equipment and Technologies Health Service Quality and Equity
3	Beyond carrots and sticks. Exploring faculty motivation to join a digital health professions educator program	Schumann et al. (28)	2025	Qualitative Study	Germany	This study aims to explore the motivation of teaching health professionals to voluntarily participate in the Digital Health Professions Education (d-HPE) program, a 200-h certification program at the Charité—Universitätsmedizin Berlin to train digital teaching skills and competencies.	Communication and Collaboration Content Creation	Public Health Workforce Development
4	Telehealth acceptance and perceived barriers among health professionals: pre-post evaluation of a web-based telehealth course	Rettinger et al. (35)	2025	Interventional Study	Austria	This study aimed to evaluate the impact of a web-based telehealth training course on health care professionals’ telehealth acceptance and their perceived barriers to telehealth adoption.	Communication and Collaboration Problem Identification and solving	Public Health Emergency Management, Access to and Utilization of Health Products, Supplies, Equipment and Technologies
5	Training gaps in digital skills for the cancer health care workforce based on insights from clinical professionals, nonclinical professionals, and patients and caregivers: qualitative study	Liñares et al. (29)	2025	Qualitative Study	European Union	The aim of this work is to identify training gaps and prioritize the digital skill development needs in the oncology health care workforce.	Communication and Collaboration Problem Identification and solving	Public Health Workforce Development
6	Bridging the AI-literacy gap in health care: qualitative analysis of the Flanders case study	Chatzichristos et al. (30)	2025	Qualitative Analysis	Belgium	While addressing key research questions, the study investigates the necessary prerequisites, barriers, and 7opportunities for AI adoption and specific training priorities that medical staff require. The study is uniquely focused on the health care workforce, moving beyond the predominant emphasis in the literature on medical students.	Communication and Collaboration	Public Health Workforce Development
7	The management of digital competence sharing in health care: a qualitative study of managers’ and professionals’ views	Hammarén et al. (31)	2024	Qualitative Study	Finland	To describe managers’ and professionals’ views on the management of digital competence sharing in health care.	Communication and Collaboration	Public Health Workforce Development
8	Digital adaptability competency for healthcare professionals: a modified explorative e-Delphi study	Bleijenbergh et al. (32)	2023	Qualitative Study	Belgium	To establish items of the digital adaptability competency for healthcare professionals.	Communication and Collaboration	Public Health Workforce Development
9	Identification of factors influencing the adoption of health information technology by nurses who are digitally lagging: in-depth interview study	De Leeuw et al. (33)	2020	Qualitative Study	The Netherlands	Our primary research objective was to identify factors that influence the adoption of HIT in a sample of nurses who describe themselves as digitally lagging behind the majority of their colleagues in their workplaces. Furthermore, we aimed to formulate recommendations for practice and leadership on how to help and guide these nurses through ongoing digital transformations in their health care work settings.	Communication and Collaboration	Public Health Workforce Development
10	Teaching digital health at Sorbonne University: the year one review of the SN@SU project	Bigay et al. (25)	2025	Quantitative Study	France	The French healthcare system faces challenges including an aging population, chronic diseases, medical staff shortages, and global pandemics, highlighting the need for a more sustainable model. At the same time, technological advances are reshaping healthcare, leading France to launch the “Digital Health Acceleration Strategy” under the France 2030 plan, aimed at equipping future health professionals as well as students in computer sciences and engineering with comprehensive digital health skills. Education plays a vital role, as shown by the SN@SU project led by Sorbonne University. This five-year initiative (2023–2028) aims to train 35,000 students in health, paramedical, and IT fields through partnerships with 17 institutions. In its first year, over 4,000 students were trained using innovative approaches such as virtual simulations, immersive environments, and e-learning. While younger students valued practical sessions, advanced students reported limited hands-on experience. Early feedback reflects both excitement about digital health learning and the need for better integration into curricula, underscoring the project’s potential to shape digital health education.	Information Search, evaluation and management, Communication and Collaboration	Public Health Workforce Development
11	Digital competencies for nurses: tools for responding to spiritual care needs	Burgos et al. (36)	2022	Qualitative Study	Spain	This article shows the results of qualitative research applying as a research tool an open-ended questionnaire, which allows detecting the educational needs for nurses’ interventions aimed at providing spiritual support to their patients using digital tools. The results obtained reveal that nurses need education and training on fundamental spiritual concepts and digital competencies to meet the multiple demands of their patients’ spiritual needs. Finally, we present an open digital educational proposal for the development of competencies for nurses and other health professionals to provide spiritual care with the support of digital tools.	Communication and Collaboration	Access to and Utilization of Health Products, Supplies, Equipment and Technologies Health Service Quality and Equity
12	Design, implementation, and analysis of an assessment and accreditation model to evaluate a digital competence framework for health professionals: mixed methods study	Saigí-Rubió et al. (26)	2024	Mixed-Method Study	Spain	This study had two objectives: (1) to create a specific map of digital competences for health professionals and (2) to define and test a digital competence assessment and accreditation model for health professionals.	Communication and Collaboration Information search, evaluation and management	Public Health Workforce Development

## 3 Results

Following the screening process, 12 studies were included in the evidence gap analysis. The included studies were published between 2020 and 2025 with a primary focus on healthcare professionals. Of the 12 included studies, eight employed qualitative designs, two used mixed-methods approaches, and two were quantitative, one of which was an interventional study. Geographically, the evidence base covered nine individual countries, while one study had a European focus. The data extraction table (Table 6) provides a structured overview of the evidence base across dimensions such as competence type, public health function, study type, and geographic context.

The evidence was concentrated in a limited number of matrix areas. “Public Health Workforce Development” was represented across all DigiComp dimensions except “Safety, Wellbeing and Responsible Use “and included 9 studies in total (25–33), with the largest cluster identified at its intersection with “Communication and Collaboration” (*n* = 9) (25–33). “Access to and Utilization of Health Products, Supplies, Equipment and Technologies” was addressed by four studies (*n* = 4) (27, 34–36), mainly in relation to “Communication and Collaboration” (*n* = 4) (27, 34–36) and “Problem Identification and Solving” (*n* = 3) (27, 34, 35). In addition, two studies (*n* = 2) were mapped to “Health Service Quality and Equity” in combination with “Communication and Collaboration” (34, 36), while only one study (*n* = 1) addressed “Public Health Emergency Management” (35).

Several evidence gaps were identified across the evidence gap matrix. No studies were found at the intersections between the DigiComp dimension “Safety, wellbeing and responsible use” and the EPHFs “Public Health Surveillance and Monitoring,” “Public Health Stewardship,” “Multisectoral Planning, Financing and Management for Public Health,” “Health Protection,” “Community Engagement and Social Participation,” and “Public Health Research, Evaluation and Knowledge.” More broadly, no studies were identified for the DigiComp dimension “Safety, wellbeing and responsible use” overall, indicating a lack of evidence in this area.

Further gaps were also observed in relation to the overall distribution of evidence across the framework dimensions. The DigiComp dimension “Information search, evaluation and management” was represented in two studies (*n* = 2) (25, 26), while “Content creation” was addressed by one study (*n* = 1) (28). At the level of the EPHFs, no studies were identified for “Public Health Surveillance and Monitoring,” “Public Health Stewardship,” “Multisectoral Planning, Financing and Management for Public Health,” “Health Protection,” “Community Engagement and Social Participation,” and “Public Health Research” as well as “Evaluation and Knowledge.” Across the included studies, several cross-cutting research gaps were identified. The evidence base was predominantly qualitative, with only two quantitative studies identified.

## 4 Discussion

The evidence gap map reveals a substantial number of gaps in the existing literature, particularly regarding the breadth and depth of digital literacy in the public health workforce. The most important finding of this evidence gap analysis was the uneven distribution of research across the intersections of the DigiComp dimensions and the EPHFs. Evidence was heavily concentrated in “Public Health Workforce Development,” particularly in relation to “Communication and Collaboration,” whereas several public health functions and digital competence domains remained weakly represented or entirely unaddressed. In addition, the evidence base was methodologically narrow, being dominated by qualitative and mixed-methods studies, with only one quantitative study identified.

Several factors may explain the identified research gaps. First, digital literacy in health contexts is often approached from a technical rather than a literacy-based perspective (37, 38), which may limit the systematic investigation of digital literacy within public health functions. Second, digital training and capacity building in public health represent relatively recent developments, meaning that research in this area is still emerging. Third, the public health workforce itself is highly heterogeneous, encompassing a wide range of professional backgrounds, while public health as a scientific discipline is comparatively young. This complexity may contribute to the limited availability of structured research focusing on specific workforce groups. Furthermore, workforce-related research in public health remains generally underdeveloped (39–41). Finally, existing studies tend to focus on general workforce development (2, 5) rather than examining more specialised competency and literacy needs within specific public health functions, which may further contribute to the observed gaps.

The identified cross-cutting gaps suggest that research on digital literacy in public health is still at an early stage of development. The predominance of qualitative studies suggests that research on digital literacy in the public health workforce is at an exploratory stage. The limited use of quantitative designs may hinder the development of comparable and quantified evidence on workforce competencies and training needs. Furthermore, the heterogeneity of digital literacy concepts, combined with the diversity of professions within the public health workforce, may currently limit the feasibility of quantitative assessments across different workforce groups. The small number of intervention studies further suggests that the implementation of training programmes in digital literacy remains under-researched, as does their impact on workforce development and practice.

The included studies also showed a geographical concentration in Western and Central Europe, highlighting the limited geographical scope of the existing evidence base. Together, these patterns indicate that the field is still evolving and requires greater conceptual clarity, methodological diversification and broader geographical representation.

### 4.1 Future research

Future research is needed to address the identified gaps by expanding the focus to DigiComp dimensions and EPHFs, particularly by examining their intersections as a guiding structure for competency development. Further studies should also focus on strengthening the translation of existing evidence on public health workforce development into practice, for example through the adaptation of curricula and the implementation and evaluation of workforce training programmes. In addition, there is a need for the development and validation of competency assessment tools to enable more systematic evaluation of digital literacy across different public health workforce groups. Future research should also pay greater attention to the interdisciplinary nature of the public health workforce and broaden the geographical scope beyond the currently dominant Western European contexts. As the field is still at an early stage of development, research trajectories may be shaped by path dependencies, regulatory developments, and external drivers such as public health crises, as observed during the COVID-19 pandemic.

The geographic scope of this analysis was limited to the European Union, which excluded studies from non-EU countries such as the United Kingdom. This boundary was chosen to align with the EU policy context of the DigiComp framework. However, future research that includes data from public health systems in non-EU countries could provide a more nuanced evidence base considering the data base used to develop the WHO EPHF framework. In this context, it should be noted that the evidence gaps identified in this analysis reflect the body of literature retrieved through the adopted search strategy and inclusion criteria. They indicate areas that are underrepresented in the currently available peer-reviewed literature rather than the definitive absence of research. Studies not captured by the search strategy, published in languages other than English, or falling outside the defined geographic or temporal scope may exist and should be addressed in future research.

The evidence gap map revealed major inconsistencies in how digital literacy is defined and measured within the public health workforce. Although the term is often used broadly, these gaps suggest limited definitional clarity and conceptual coherence, especially in distinguishing digital competence, digital literacy, and broader information and communication technology (ICT) capabilities. This is reflected in the lack of studies examining the DigiComp framework dimensions of “Information search, Evaluation and Management,” “Safety, Wellbeing and responsible Use,” and “Problem Identification and Solving.”

### 4.2 Implications for public health workforce development and public health practice

The findings highlight several implications for public health workforce development and digital literacy strategies. First, training needs should be more clearly specified and differentiated according to the diverse professional groups within the public health workforce. The EPHFs could serve as a useful guiding framework to better align competency development with functional workforce requirements. Second, existing competency frameworks should be further expanded to address the identified gaps while building on established digital competence and literacy models. For instance, it should be noted that the WHO-ASPHER Competency Framework for the Public Health Workforce in the European Region (42) provides an additional conceptual reference point for studies in this field. While this framework was not used as a structural axis in the present analysis, its competency domains overlap with several of the EPHF functions examined here. Future evidence gap maps in this area may benefit from explicitly integrating the WHO-ASPHER framework to further contextualise findings within a European public health workforce perspective. Given that digital literacy represents a cross-cutting competence relevant across multiple sectors, future strategies should also consider foundational digital skills developed through general education, vocational training, and higher education pathways.

Furthermore, while initial workforce development programmes in digital literacy are emerging, there remains a need for more robust evaluation of their effectiveness and impact, as the current evidence suggests that research in this area is still largely exploratory. Capacity building also emerges as a key consideration, as digital transformation not only requires resources but also redistributes capacities within public health systems. This highlights the need for a better understanding of how digitalisation affects workforce structures and resource allocation.

Finally, the findings point to important policy implications. Various policy initiatives already aim to strengthen the framework conditions and resource base for public health workforce development and digital competency frameworks. However, the sustainability and long-term impact of such programmes require further attention. For example, initiatives such as the German “Pact for the Public Health Service” have invested substantial resources in digitalisation and workforce literacy, yet questions remain regarding their long-term institutionalisation, continuity and further development. These findings underline the importance of considering policy path dependencies and ensuring that training programmes and competency frameworks are designed with a medium- to long-term perspective rather than as short-term interventions.

### 4.3 Strengths and limitations

This evidence gap analysis has several strengths. To the best of our knowledge, the study at hand represents the first systematic mapping of the intersections between the EPHF and the DigiComp framework. The study followed a systematic and transparent methodological approach and integrated conceptual frameworks to structure the analysis. Furthermore, methodological rigour was strengthened using a double-screening process, which increased the reliability of study selection and classification.

However, several limitations should also be considered. The use of the EPHF and DigiComp frameworks may introduce selection bias. In addition, the conceptual overlap between digital literacy and digital competence remains unclear, raising questions about the suitability of the DigiComp framework for mapping digital literacy evidence. The relatively small number of 12 included studies limits the generalisability of the findings. The search was restricted to selected databases and to studies published in English, which may have excluded relevant evidence. Although English-language publications may reflect broader European trends, this approach likely overlooks studies with a narrower, local focus, for instance, German-language research on digital literacy in the context of the PHS. In addition, the mapping of studies to the respective framework dimensions involved a degree of selective bias despite efforts to ensure consistency. No formal quality appraisal of the included studies was conducted, as the aim of the analysis was to map the distribution of evidence rather than to assess study effectiveness. Moreover, given the rapidly evolving nature of digital health and digital literacy research, the findings represent only a snapshot of a dynamic field. Finally, the analysis did not assess the effectiveness or outcomes of the included studies, as the focus was on identifying the presence or absence of research rather than synthesising study results.

Despite this study’s limitations, this evidence gap analysis revealed substantial trends in the current European literature on digital literacy of public health workforces and contributes to the dynamic field of digital public health by informing future research priorities and supporting evidence-based workforce development.

## 5 Conclusion

In conclusion, the available evidence on digital literacy in the public health workforce is unevenly distributed, with clear concentration areas as well as substantial gaps across both the DigiComp dimensions and the EPHFs. The evidence base is also geographically concentrated and remains largely exploratory in nature. Future research should prioritise the synthesis and refinement of existing competency frameworks, pay greater attention to specific public health professions through the lens of the EPHFs, and strengthen the transfer of existing evidence into practice through the implementation and quantitative evaluation of training and intervention approaches. The evidence gaps shown in this study underline the acknowledgment of the systemic nature of digital literacy, which cannot be reduced to isolated skill-sets or individual capacities. Rather, digital literacy must be understood as a cross-cutting structural enabler of successful digital transformation in public health systems and their EPHFs. It is not only a question of individual upskilling and workforce development, but also of institutional readiness and strategic alignment across all levels of the public health sector.

The COVID-19 pandemic may have acted as a catalyst for digitalisation, yet it also revealed structural weaknesses and inequalities. While some public health professionals are expected to be able to adapt rapidly, others were left behind or faced overwhelming challenges. This underscores the importance of designing inclusive, long-term strategies for capacity building and digital preparedness of the public health workforce in Europe.
